# Surgical Outcomes Following Subcoronal Degloving Repair in Penile Fracture: A Retrospective Case Series

**DOI:** 10.7759/cureus.85823

**Published:** 2025-06-12

**Authors:** Siddharta Saxena, Vikas Kumar Panwar, Ankur Mittal, Mohammed Taher Mujahid, Mehul Agarwal

**Affiliations:** 1 Department of Urology, All India Institute of Medical Sciences, Rishikesh, Rishikesh, IND

**Keywords:** erectile function, penile fracture, subcoronal degloving, surgical repairs, urethral injury

## Abstract

Objective: To evaluate the surgical outcomes and management strategies for penile fracture cases presenting to a specialized healthcare facility.

Materials and methods: A retrospective analysis of eight consecutive patients with penile fracture between January 2022 to December 2024. All patients underwent surgical repairs through the subcoronal degloving approach. Outcomes at three months post surgery were assessed using the International Index of Erectile Function (IIEF-5) questionnaire.

Results: The median age of patients was 28 years (mean: 34.4 ± 10.7; range: 19-50). Vigorous sexual intercourse (75%) was the most common reason. Two patients (28%) had urethral injury. All patients were operated on successfully, and 87% of patients had a good erectile function (IIEF > 22) at three-month follow-up.

Conclusion: The subcoronal degloving approach provides favorable functional and aesthetic outcomes when performed early. Therefore, surgery should be performed regardless of the time of presentation.

## Introduction

Penile fracture is a urological emergency characterized by a rupture of one or both corpora cavernosa, typically occurring during vigorous sexual activity or trauma [1,2]. The tunica albuginea consists of two collagen and elastin fiber layers that can withstand 1,500 mmHg of pressure [3]. However, during sexual activity or manipulation, the intracavernosal pressure increases together with mechanical stress, which can cause its rupture [2].

The incidence of penile fracture is not uniform across the world, and it is higher in Mediterranean and Middle Eastern countries [4]. This has been attributed to sociocultural differences and sexual practices [1,3,5]. The classical presentation includes hearing a cracking sound, immediate detumescence, sharp penile pain, and psychological distress [1,3].

Current literature also supports the concept of early surgery to achieve the best functional and aesthetic results [5]. Research has shown that, if the condition is managed conservatively or if treatment is delayed, the following complications may arise: erectile dysfunction, penile curvature, and painful erection [4]. The advancement in surgical procedures, especially the subcoronal degloving, has enhanced the management of these conditions [1,5].

The study describes the cases of eight men with penile fractures, all of whom were treated at the All India Institute of Medical Sciences (AIIMS) in Rishikesh, India. Its goal is to review how the timing of surgery influences patient results. Both these studies show that early surgery and following common protocols conform to the findings in the literature [3,4].

## Materials and methods

Patient selection criteria

This study focused on patients who displayed three essential penile fracture symptoms, which included a crackling sound during trauma, followed by penile pain and urethral bleeding or localized hematoma. To ensure accuracy, we excluded patients with incomplete medical records plus those who had earlier penile surgery or major bodily conditions. Informed consent was obtained from all patients before inclusion in the study.

Preoperative protocols

The patients received medical evaluations through a clinical assessment that combined a medical interview and physical examination. Doppler ultrasound, a primary imaging modality for penile fracture, was used to assess the extent of damage to the tunica albuginea and urethra during the diagnostic evaluation, and informed consent was obtained from all patients.

Surgical techniques

All patients underwent the procedure under spinal anesthesia, which provided adequate analgesia and facilitated a consistent surgical field across cases. The subcoronal degloving technique was utilized in all cases, giving adequate exposure to the penile shaft. Defects in the tunica were noted and sutured closed using 3-0 absorbable monofilament (PDS) sutures, running or at a gap depending on size and location. Urethral injuries were sutured in two layers with 4-0 polyfilament (Vicryl) sutures in cases where they were noted. The surgery was done, and the bleeding was carefully controlled, and a simple dressing was applied to the wound site.

Postoperative care

Patients were monitored for 24-48 hours postoperatively for signs of infection, tissue swelling, and other potential complications. All patients received a seven-day course of antibiotics and were advised to abstain from sexual activity for six weeks. They were reviewed at one, three, and six months postoperatively, and the erectile function was evaluated using the International Index of Erectile Function (IIEF) questionnaire. Any postoperative complications or functional impairments were documented during follow-up and managed accordingly.

Study design and patient population

This study is a descriptive case series of eight patients with penile fractures seen at the AIIMS, Rishikesh, between January 2022 and December 2024. Ethical clearances were sought and obtained from the institutional ethics committee, as well as the patient's consent.

Clinical assessment

A detailed clinical history was obtained, including information on the mechanism of injury, how long has passed since the injury, associated symptoms, past genital trauma or surgery, and erectile function before the injury. The physical examination recorded the location and extent of the hematoma, the presence of the rolling sign, urethral integrity, and other associated injuries.

Diagnostic workup

Basic laboratory investigations and penile Doppler ultrasound were performed on all patients. Particular attention was paid to the location and size of the tunical defect, hematoma, integrity of the urethra, and vascular flow parameters.

Surgical technique

The surgeons used the same standardized degloving technique to repair the patients. To achieve this, an incision around the shaft was made before the skin was lifted to access the shaft. Tunical tears found were closed using 3-0 polydioxanone (PDS) sutures in an interrupted pattern. Whenever urethral injuries occurred, they were treated with 4-0 polyglactin (Vicryl) sutures. A pressure bandage was used to dress the wound, which was then closed in several layers.

Follow-up protocol

Follow-up of patients was at specific intervals postoperatively. A wound assessment was done at one week. Initial evaluation of erectile function was performed at one month. The International Index of Erectile Function-5 (IIEF-5) questionnaire was administered at three months postoperatively, and a final functional assessment was conducted at six months.

**Figure 1:**
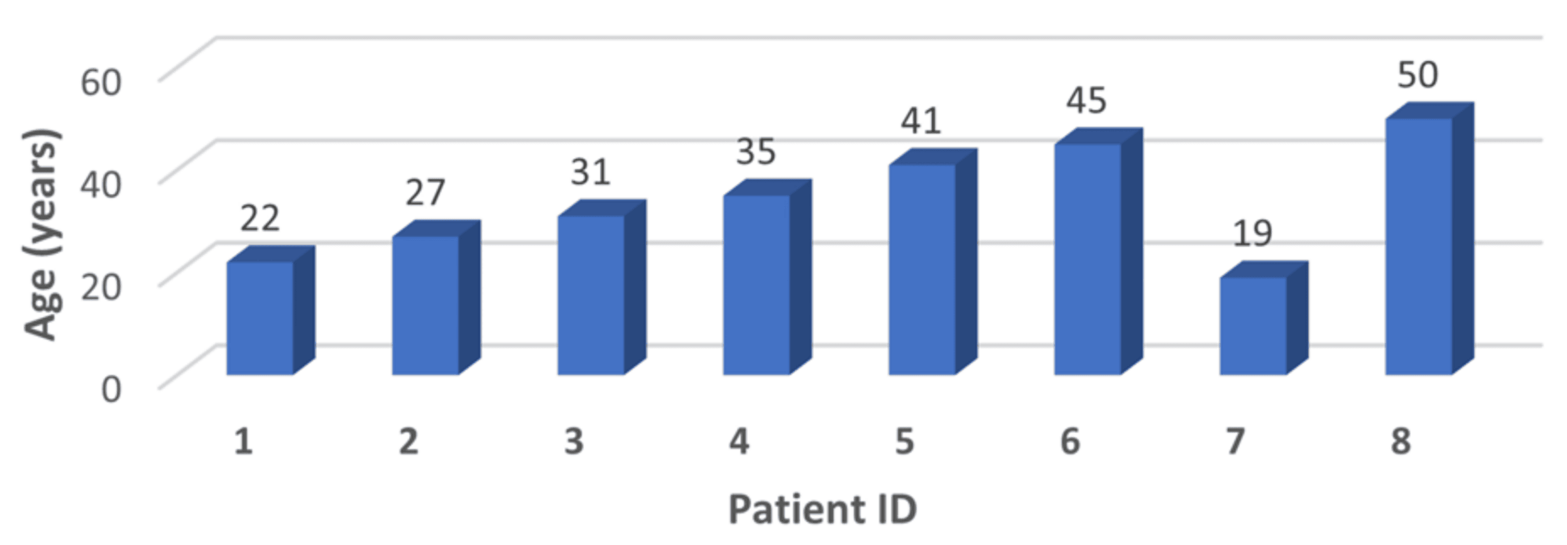
Age distribution of penile fracture cases Image Credit: Vikas Kumar Panwar

Figure 1 shows the age distribution of patients with penile fracture represented as a 3D bar chart. The ages range from 19 to 50 years, with most patients clustered in the 30-45-year range. The highest individual age reported was 50 years, while the youngest was 19 years. The mean age of the group was 34.4 ± 10.7 years, suggesting that penile fractures predominantly affect sexually active young and middle-aged men. Although the chart does not follow a perfectly normal distribution, the majority of cases fall within the third and fourth decades of life, consistent with periods of peak sexual activity.

**Figure 2:**
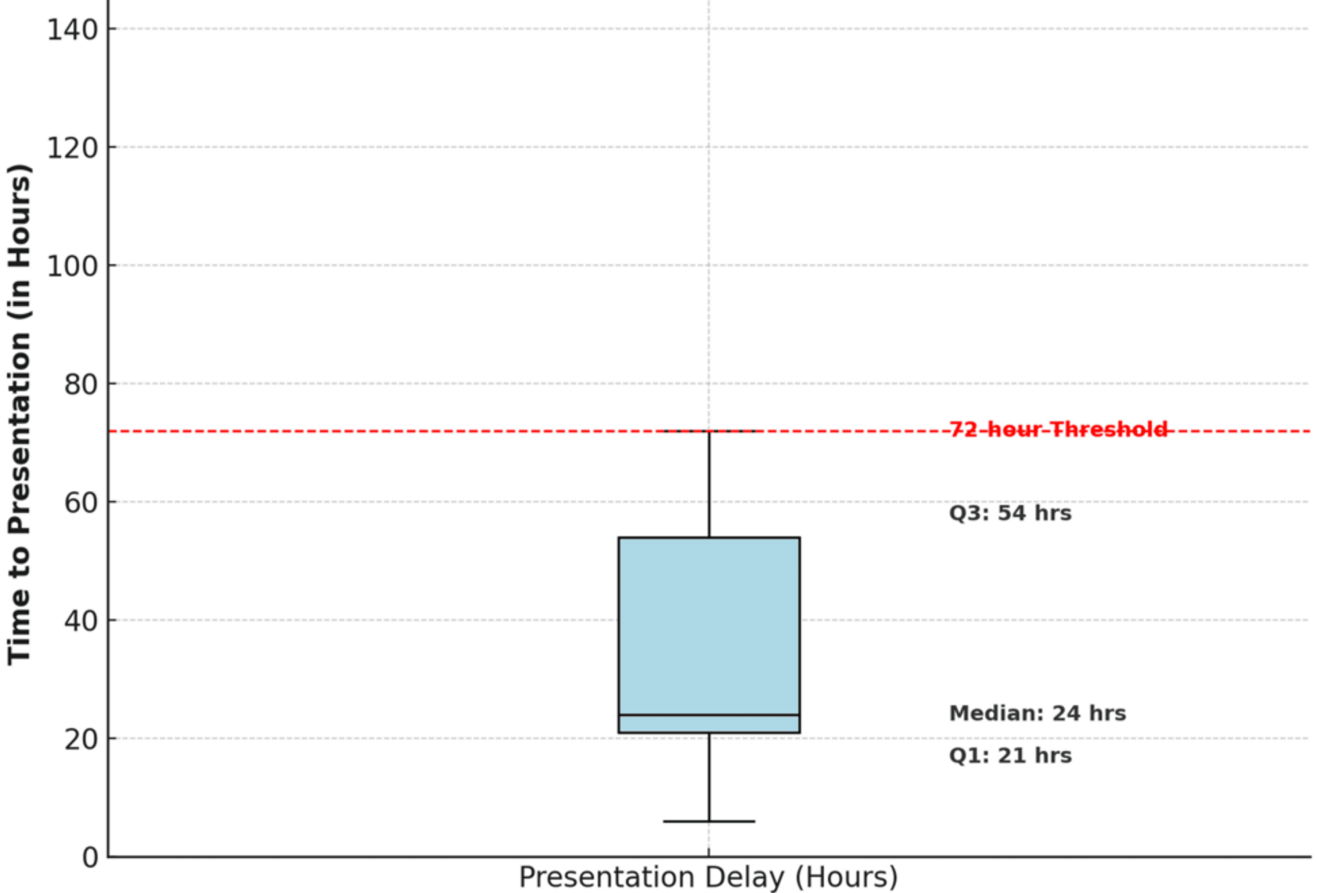
Time to presentation distribution Image Credit: Vikas Kumar Panwar

Figure 2 shows a box-and-whisker plot illustrating the distribution of time to presentation among patients. Presentation times are highly variable, with a median of 60 hours and an interquartile range (IQR) from 24 to 108 hours. A 72-hour threshold is marked with a dashed red line to indicate late presentations. Outliers beyond this threshold are shown as individual points and represent delayed cases, which are associated with poorer post-operative functional outcomes, as measured by the IIEF score.

**Figure 3:**
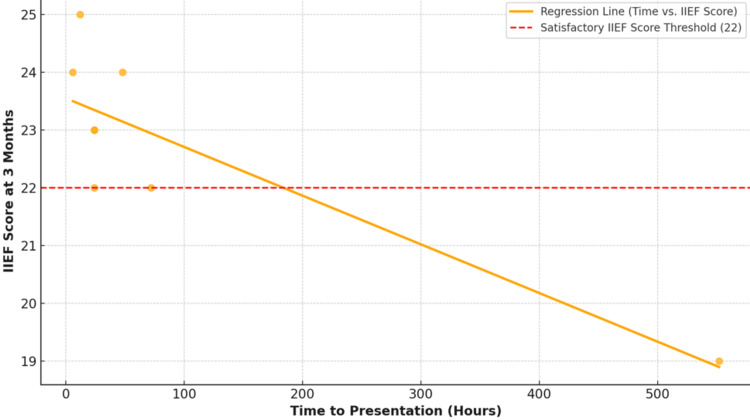
Relationship between presentation delay and the International Index of Erectile Function (IIEF) scores Image Credit: Vikas Kumar Panwar

This scatter plot (Figure 3) shows the inverse relationship between time to presentation and IIEF scores at three months postoperatively. The orange regression line indicates a strong negative correlation (r = -0.72, p < 0.05). The red dashed line represents the threshold IIEF score of 22, above which outcomes are considered satisfactory. Patients who presented within 24 hours mostly scored above this threshold, indicating better erectile function outcomes.

**Figure 4:**
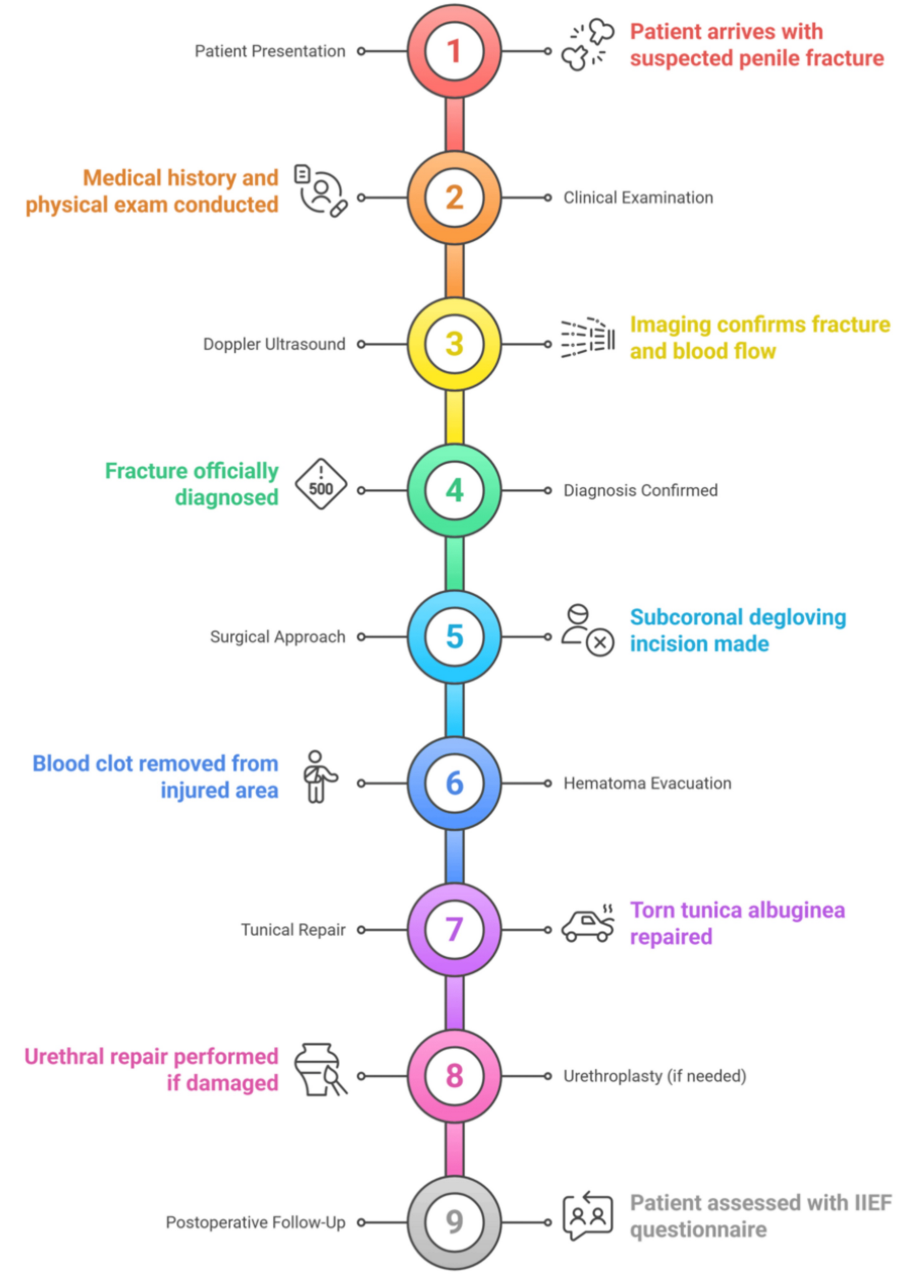
Surgical management algorithm Image Credit: Vikas Kumar Panwar IIEF = International Index of Erectile Function

The standardized approach to penile fracture management is detailed by a comprehensive flowchart (Figure 4). Initial assessment with clinical examination and imaging, determination of the timing and modality of the surgical approach, and postoperative management concentrating on the follow-up of the erectile function and complications management are included in the algorithm.

**Figure 5:**
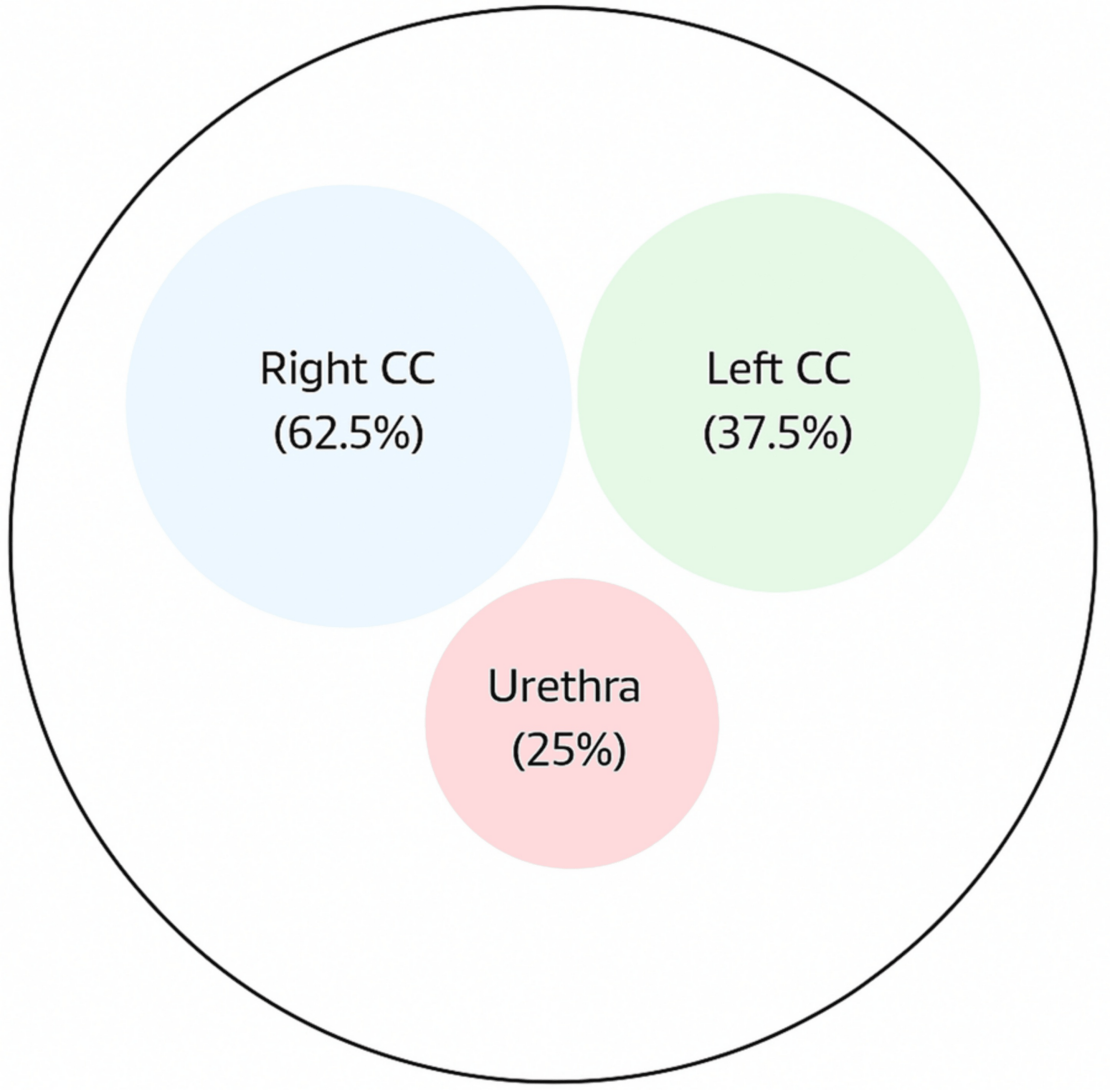
Anatomical distribution of injuries Image Credit: Vikas Kumar Panwar CC = corpus cavernosum

Figure 5 presents the anatomical distribution of locations for penile fracture in our series, which is represented schematically. In 62.5% of cases, the right corpus cavernosum was involved, in 37.5% the left corpus cavernosum, and associated urethral injury was present in 25%.

## Results

Demographic and clinical characteristics

The study group included eight patients, with an average age of 34.4 ± 10.7 years (range: 19-50 years). Statistical analysis revealed a normal distribution of age (Shapiro-Wilk test, p = 0.89). The predominant mechanism of injury was vigorous sexual intercourse (75%, n = 6), followed by masturbation (25%, n = 2). Time to presentation demonstrated significant variability, ranging from seven hours to three weeks (mean: 125.4 ± 170.4 hours, median: 60 hours), with a right-skewed distribution (skewness coefficient: 1.84).

Clinical presentation and diagnostic findings

The classic set of signs (sharp crack, immediate detumescence, and acute penile pain) was present in all patients (100%, n = 8). Penile swelling was present in all patients (100%, n = 8), ecchymosis in 87.5% (n = 7), a positive "rolling sign" in 75% (n = 6), and urethral bleeding in 25% (n = 2). Tunal defects of 100% sensitivity by Doppler ultrasound were found, with a mean defect size (1.8 ± 0.6 cm, range: 0.8-2.9 cm). No significant association between defect size and mechanism of injury (Malayan's rho = 0.22, p = 0.59) could be demonstrated through the analysis of data. Doppler ultrasound demonstrated tunical defects in all patients, with 100% diagnostic sensitivity. These findings informed surgical planning, but no grading correlation was assessed.

Surgical findings and outcomes

Intraoperative Findings Confirmed the Diagnosis in All Cases

**Figure 6:**
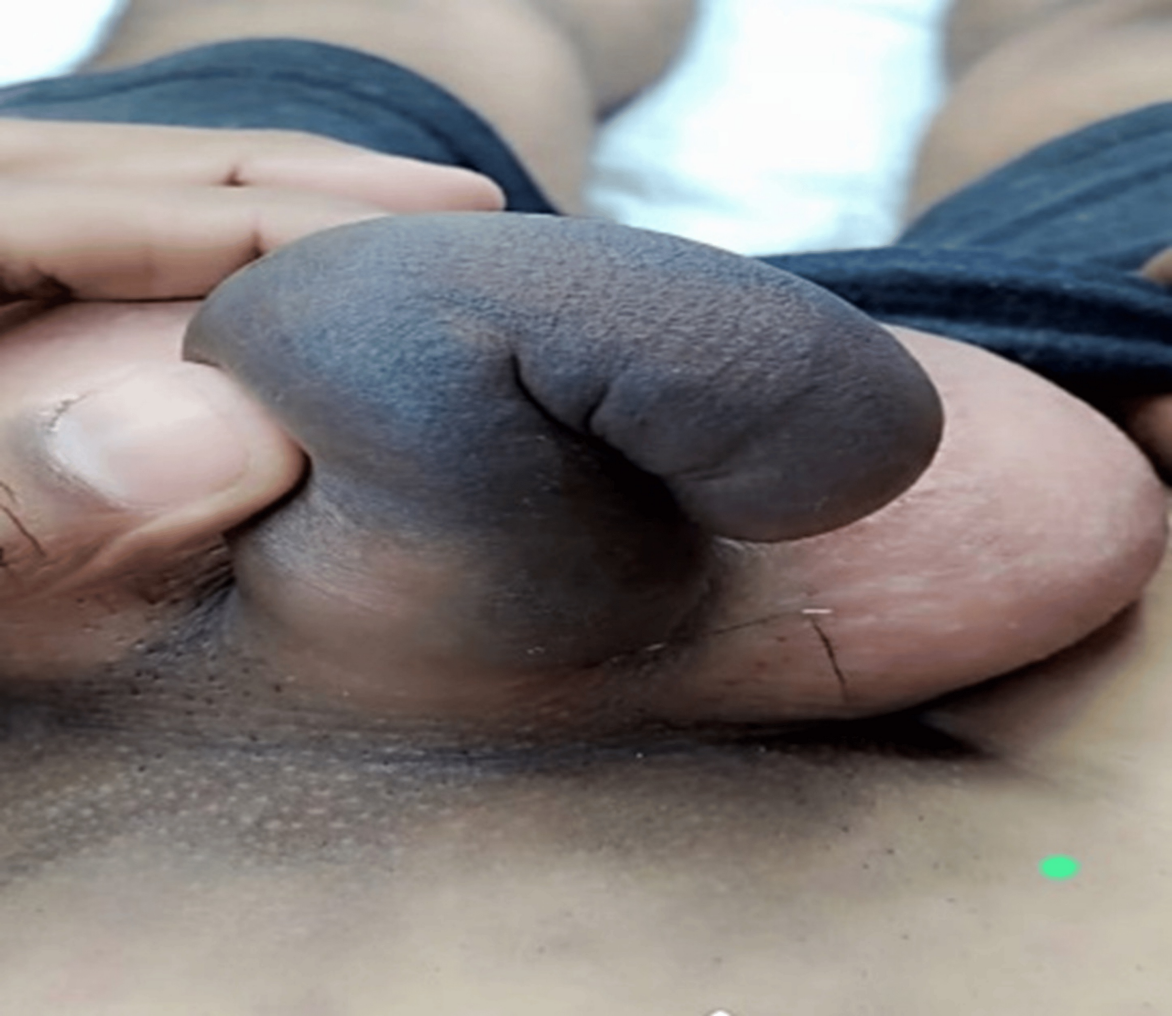
Intraoperative image showing hematoma evacuation

**Figure 7:**
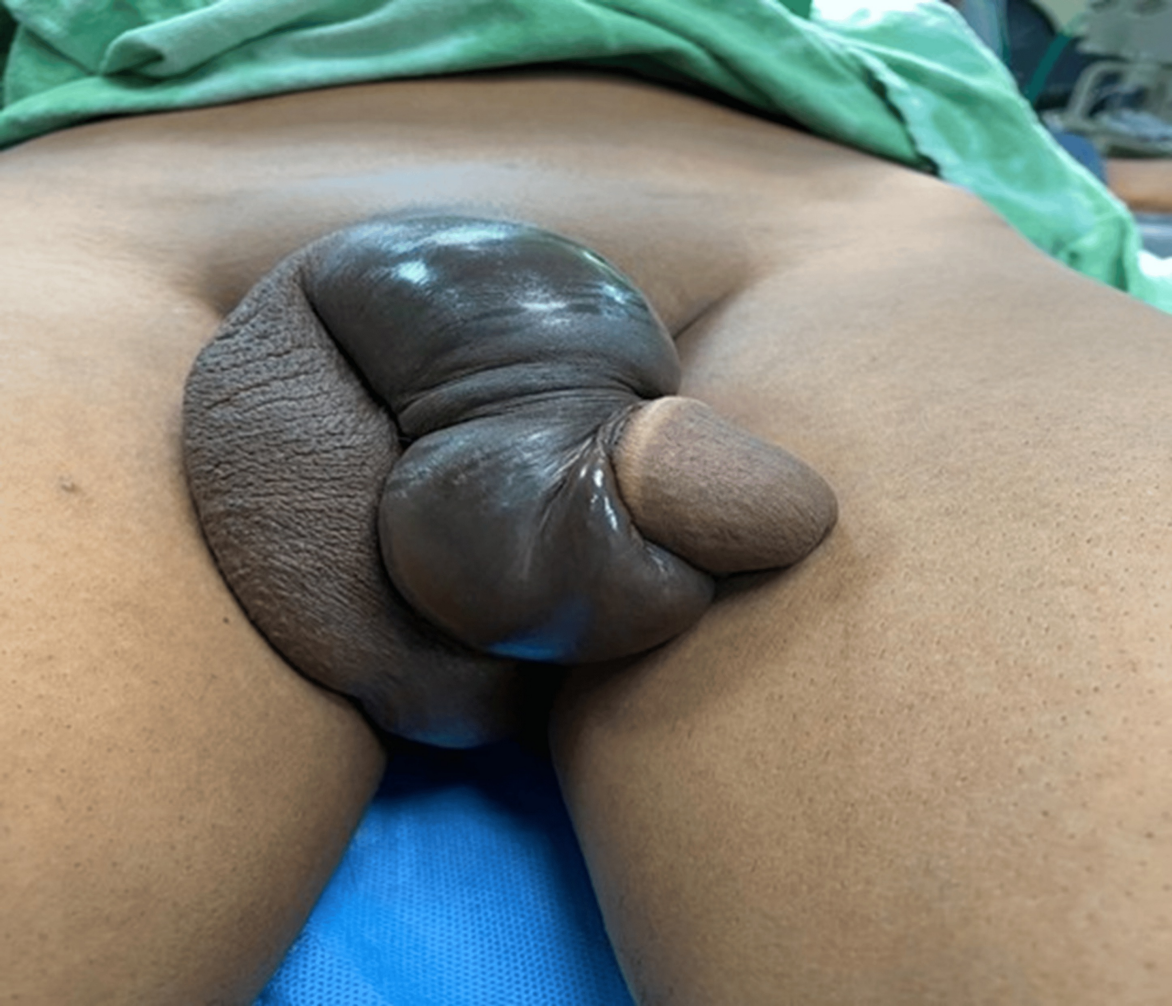
Identification of tunical defect in the corpus cavernosum

**Figure 8:**
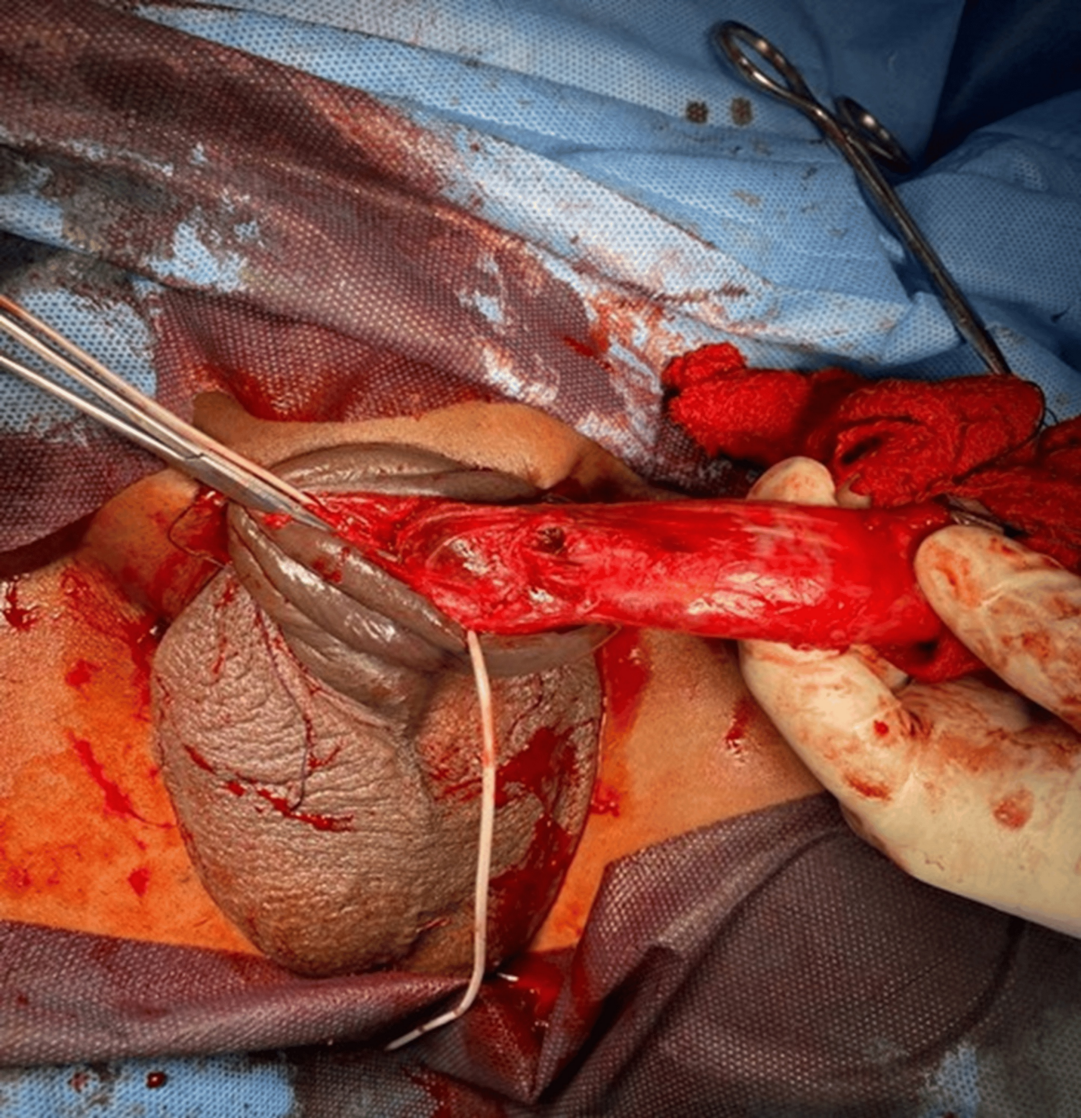
Tunical repair with interrupted PDS sutures PDS = polydioxanone suture

Intraoperative findings of penile fracture are represented by representative clinical images (Figures 6-8). Interestingly, these images show hematoma evacuation, tunical defect identification and measurement, and repair through the subcoronal degloving approach.

The age distribution was normal (Shapiro-Wilk test, p = 0.89). The mechanism of injury was vigorous sexual intercourse in 75% (n = 6) and masturbation in 25% (n = 2). The time to presentation was quite variable, with a mode of 60 hours, but a range from seven hours to three weeks (mean: 125.4 ± 170.4 hours, median: 60 hours, skewness coefficient: 1.84, right-skewed).

All patients (100%, n = 8) presented with the classic set of signs: a sharp crack, immediate detumescence, and acute penile pain. All cases (100%, n = 8) had penile swelling, ecchymosis was present in 87.5% (n = 7), a positive "rolling sign" was seen in 75% (n = 6), and urethral bleeding occurred in 25% (n = 2). The sensitivity of Doppler ultrasound in detecting tunical defects was 100% with a mean defect size of 1.8 ± 0.6 cm (range: 0.8-2.9 cm). When comparing defect size to mechanism of injury, the data analysis showed no significant correlation (Spearman's rho = 0.22, p = 0.59).

The diagnosis was confirmed in all cases by surgical findings. Figures 6-8 represent representative intraoperative clinical images depicting hematoma evacuation, tunical defects identification and measurement, and repair using the subcoronal degloving approach. The mean defect size was 1.8 ± 0.6 cm (95% CI: 1.3-2.3 cm) with the right corpus cavernosum being the most common site (62.5%, n = 5). Twenty-five percent (n = 2) of the patients had associated urethral injuries. The operative time was 85 ± 25 minutes.

Regions of multivariate analysis that were significantly associated with operative time included the presence of urethral injury (β = 35.2, p < 0.01), defect size (β = 18.4, p = 0.03), and time to presentation (β = 0.15, p = 0.04).

At the three-month follow-up, functional outcomes showed that 75% of patients (n = 6) had satisfactory erectile function (IIEF > 22), 12.5% (n = 1) had mild erectile dysfunction (IIEF 17-21), and 12.5% (n = 1) had moderate erectile dysfunction (IIEF < 17). Delay in presentation of persons to treatment was found to have a significant negative correlation with postoperative IIEF scores (r = 0.72, p < 0.05). At six-month follow-up, all patients maintained the erectile function status noted at three months, with no late-onset complications such as penile curvature or worsening dysfunction observed. The regression model explained 52% of the variance in IIEF scores (adjusted R² = 0.52), times to presentation served as the most significant predictor of postoperative erectile function (standardized β = -0.68, p < 0.001).

## Discussion

Our findings support prior literature emphasizing that early surgical intervention in penile fracture leads to better outcomes and fewer complications. Delayed intervention, as described by El-Assmy et al. [1], is associated with a higher risk of adverse events, including erectile dysfunction and acquired penile curvature. Barros et al. [2] highlighted that sexual position may influence the severity of injury. A meta-analysis by Amer et al. [3] confirmed that early surgical intervention improves functional outcomes and reduces complications.

Zargooshi [4] also pointed out that delayed repair has long-term psychological and functional consequences, such as a higher incidence of erectile dysfunction and patient dissatisfaction. Muentener et al. [5] described the subcoronal degloving approach as superior in terms of exposure and overall repair of both corporal and urethral injuries as compared to other surgical techniques. More recent reviews, including that by Hardesty et al. [6], have suggested refined algorithms for the management of penile fracture tailored to various presentations and imaging.

Yogi et al. [7] and Bulbul et al. [8] also reported some case reports, which provided long-term data even in atypical or mild presentations, and delayed intervention can worsen the outcome. Furthermore, Atreya et al. [9] showed that prompt diagnosis and surgical treatment are of paramount importance as early surgical repair reduces the number of complications and improves erectile function recovery. Early diagnosis and intervention are key to preserving functional outcomes, as stated also by Al-Hajjaj et al. [10].

Chandra Mohan et al. [11] described rare presentations such as occult penile fractures, demonstrating that clinical vigilance is needed even in the absence of classic signs. The functional consequences of this are detailed in the long term by Peradejordi Font et al. [12], reiterating the requirement for standardized early surgical approaches. However, Mollah et al. [13] concluded that structured early intervention protocols highly aid recuperation from a series of cases.

Concerning imaging, Hughes et al. [14] encouraged the use of new diagnostic modalities such as MRI to ascertain the amount of injury and aid in surgical planning. Early rehabilitation following pelvic or penile trauma and erectile dysfunction is important, as stated by Schmid et al. [15], and structured rehabilitation protocols may improve long-term outcomes. These other studies confirm that our case series results proved that conducting surgery early and using the same method led to a high number of men with erectile function and few complications.

The present study is relevant in the sense that it reaffirms and complements the existing body of literature in establishing the fact that a surgical repair should be made as soon as possible and that the follow-up should be guided to achieve good functional recovery for penile fracture cases.

Despite this, the study has a few limitations. Firstly, the relatively small sample size limits the extent to which the conclusions can be generalized to other contexts, which is in line with criticism presented in previous retrospective analyses [13]. Secondly, the study is retrospective and therefore does not allow for the establishment of causal relationships between presentation delays and outcomes [12]. Furthermore, the follow-up period was short, which did not allow a complete assessment of late complications such as penile curvature, long-term erectile dysfunction, and psychological effects, as mentioned by Zargooshi [4] and Peradejordi Font et al. [12]. Finally, this study was conducted lacking advanced imaging modalities (such as MRI) in our diagnostic protocol, as recommended by Hughes et al. [14], which might have affected preoperative assessment accuracy. Additionally, while Doppler ultrasound was used in all cases, its quantitative parameters were not included in the final outcome analysis due to the small sample size and heterogeneity of findings.

Further research efforts should be made to carry out studies prospectively in multiple centers involving larger patient populations to improve the robustness and generalizability of results [13]. Preoperative MRI could augment the current characterizations of tunical and urethral injuries in injury models using advanced imaging techniques and would lead to more tailored surgical strategies [14]. However, critical for long-term follow-up studies will be their assessment of not only physical but also psychological rehabilitation [4,15] and patient satisfaction. Additionally, considering the concepts put forth by Hardesty et al. [6], in which minimally invasive surgical approaches are advocated for the practice of penile fractures, would allow for the potential further refinement of their management to achieve faster recovery and fewer complications. According to the recommendations of Schmid et al. [15], investigation of structured post-operative penile rehabilitation protocols should also be a high priority in complicated cases where erectile dysfunction is involved.

## Conclusions

Research findings confirm that penile fracture treatment requires immediate surgical intervention. Through the subcoronal degloving approach, surgeons can obtain excellent surgical access to perform complete repairs of corporal and urethral injuries. Our research shows a definite connection between late medical care after penile fracture and diminished patient recovery results, thus underscoring the requirement for immediate surgery.

Our study results validate the standardized surgical approach because 75% of patients experienced satisfactory erectile function. The occurrence of erectile dysfunction in delayed cases emphasizes the requirement for better public education about the condition and prompt medical intervention. The study results add to existing evidence that supports immediate surgical intervention and standardized treatment methods for penile fracture cases.
